# Non-Esterified Fatty Acids Generate Distinct Low-Molecular Weight Amyloid-β (Aβ42) Oligomers along Pathway Different from Fibril Formation

**DOI:** 10.1371/journal.pone.0018759

**Published:** 2011-04-19

**Authors:** Amit Kumar, Rebekah L. Bullard, Pritesh Patel, Lea C. Paslay, Dipti Singh, Ewa A. Bienkiewicz, Sarah E. Morgan, Vijayaraghavan Rangachari

**Affiliations:** 1 Department of Chemistry and Biochemistry, University of Southern Mississippi, Hattiesburg, Mississippi, United States of America; 2 School of Polymers and High Performance Materials, University of Southern Mississippi, Hattiesburg, Mississippi, United States of America; 3 Department of Biomedical Sciences, College of Medicine, Florida State University, Tallahassee, Florida, United States of America; Boston University School of Medicine, United States of America

## Abstract

Amyloid-β (Aβ) peptide aggregation is known to play a central role in the etiology of Alzheimer’s disease (AD). Among various aggregates, low-molecular weight soluble oligomers of Aβ are increasingly believed to be the primary neurotoxic agents responsible for memory impairment. Anionic interfaces are known to influence the Aβ aggregation process significantly. Here, we report the effects of interfaces formed by medium-chain (C9–C12), saturated non-esterified fatty acids (NEFAs) on Aβ42 aggregation. NEFAs uniquely affected Aβ42 aggregation rates that depended on both the ratio of Aβ:NEFA as well the critical micelle concentration (CMC) of the NEFAs. More importantly, irrespective of the kind of NEFA used, we observed that two distinct oligomers, 12–18 mers and 4–5 mers were formed via different pathway of aggregation under specific experimental conditions: (*i*) 12–18 mers were generated near the CMC in which NEFAs augment the rate of Aβ42 aggregation towards fibril formation, and, (*ii*) 4–5 mers were formed above the CMC, where NEFAs inhibit fibril formation. The data indicated that both 12–18 mers and 4–5 mers are formed along an alternate pathway called ‘off-pathway’ that did not result in fibril formation and yet have subtle structural and morphological differences that distinguish their bulk molecular behavior. These observations, (*i*) reflect the possible mechanism of Aβ aggregation in physiological lipid-rich environments, and (ii) reiterate the fact that all oligomeric forms of Aβ need not be obligatory intermediates of the fibril formation pathway.

## Introduction

Alzheimer’s disease (AD) is a progressive neurodegenerative disorder that leads to cognitive decline and memory impairment. As in many other neurodegenerative diseases, AD is one in which polypeptides form amyloid deposits. Brains of patients with AD have large proteinacious deposits known as senile plaques. The proteinacious core of these extracellular deposits is mainly composed of 40- and 42-residue peptides (Aβ40 and Aβ42, respectively), collectively called Aβ peptides. According to the early amyloid hypothesis, fibrillar Aβ was thought to be largely responsible for the neuronal dysfunction and cognitive decline in patients with AD [1]. However, the degree of cognitive impairment did not correlate well with the amount of plaque deposits prior to death in some AD patients [2], [3], while correlations between soluble Aβ levels and the extent of synaptic loss and cognitive impairment were more pronounced [4], [5]. This has led to the shift in focus towards smaller oligomeric intermediates in the aggregation pathway that may be responsible for toxicity. Specifically, smaller, low-molecular weight oligomers have been the most scrutinized as several reports have implicated them in neuronal toxicity and synaptic dysfunction. Consequently, several oligomeric species ranging between 2 and 12 mers were identified in the cerebrospinal fluid (CSF) of AD patients [5], in transgenic mouse models that express the APP variant linked to AD [6]–[8] and in neuronal cell cultures [9].

In the context of soluble oligomers, interfacial aggregation of Aβ in the presence of anionic surfactants, such as lipids, fatty acids and surfactants seem to play significant roles. Several reports demonstrated the effects of anionic phospholipids on Aβ aggregation and showed that the interaction of Aβ with lipids is restricted to the polar head groups [10]–[12]. In addition, GM1 ganglioside containing membranes have been shown to promote Aβ aggregation *in vitro* [13]–[18]. Similarly, polyunsaturated (PUFAs) as well as saturated fatty acids are also known to have significant effects on the AD brain [19], [20]. More importantly, these interfaces play unique roles in generating oligomeric forms of Aβ. In the presence of GM1 containing liposomes Aβ40 generated oligomers that were able to act as exogenous ‘seed’ in the Alzheimer’s brain [21], [22]. In addition, lipid rafts isolated from the brain tissues induced the formation of 4 mers of Aβ that did not convert to fibrils for prolonged periods of time [23], suggesting increased turnover time for these lipid-induced oligomers. A variety of physiological and non-physiological interfaces are known to influence Aβ aggregation and generation of oligomeric species [24]–[26].

One surfactant that is known to play a significant role in this regard is sodium dodecylsulphate (SDS), a commonly known detergent used for denaturing protein structures. SDS has also been demonstrated to affect the aggregation of other amyloid proteins such as β2 microglobulin and α-synuclein [27], [28], besides Aβ. Anionic micelles generated by SDS form good interface models and are observed to accelerate both Aβ40 and Aβ42 aggregation over a limited concentration range [29]. In particular, at concentrations just below its critical micelle concentration (CMC), SDS was able to promote the formation of 2–4 mers and 8–12 mers of Aβ42, but did not promote the formation of similar oligomers with Aβ40 [30], [31]. However, at concentrations well below the CMC, SDS augmented Aβ42 fibril formation rates and failed to generate oligomers. Furthermore, the 2–4 mers and 8–12 mers seemed to form from a pathway that was different from the one resulting in fibril formation described by Rangachari *et al* [30]. Interestingly, aggregates from both pathways showed a β-sheet conformation but differed in their ThT binding capability and morphology. Prior to this observation, Barghorn and colleagues also generated 38–48 kDa (8–12 mer) Aβ42 oligomers, called ‘globulomers,’ by co-incubating Aβ42 in low concentrations of SDS [32]. These exogenous globulomers were toxic and inhibited LTP in human brain slices. More importantly, the globulomers also formed independently from the fibril formation pathway, suggesting an alternate pathway induced by SDS interfaces [33]. Based on these previous reports, it is clear that by choosing appropriate concentrations of SDS as well as the SDS: Aβ42 ratio, one can induce different pathways of Aβ42 aggregation. Although SDS is a good model system to mimic anionic lipid interfaces, it is not a physiological component and hence ambiguity would remain about the significance of its interaction with Aβ. Therefore, there is a need to explore whether physiological lipids and fatty acids can dictate similar multiple pathways of Aβ aggregation and generate stable oligomers. Given the structural similarity to SDS and possible contribution to other forms of human amyloidosis[34], [35], non-esterified fatty acids (NEFAs) are important candidates for exploration of their effects on Aβ aggregation. NEFAs are produced *de novo* by adipocytes or lipolysis of plasma triacylglycerol in chylomicrons or very-low density lipoproteins, transported by serum albumin, and incorporated in adipocytes or muscle cells [36]. NEFAs are also abundant in both cerebral vasculature as well as in the CSF [37], [38], and in the brain, long-chain NEFAs (C12–C26) are abundant.

Here, we report the effects of saturated NEFAs on Aβ42 aggregation and pathways mainly to simulate Aβ-NEFA interaction under physiological conditions. These studies employed medium-chain saturated NEFAs as a model system to circumvent a solubility problem associated with long-chain NEFAs. In addition, the CMCs of long-chain fatty acids (C18–C22) are much lower (∼µM–nM) than those of their medium-chain counterparts (C9–C14; ∼mM), their interactions with Aβ at nM – pM physiological concentration range can be conveniently reproduced and examined by using medium-chain NEFAs and µM concentrations of Aβ *in vitro*. Thus, we have used fatty acids of varying carbon chain lengths including pelargonic acid (C9), capric acid (C10), undecylic acid (C11), and lauric acid (C12) to see how the CMC and Aβ : fatty acid ratios affect the overall aggregation process, particularly in dictating multiple pathways. The results indicate a fundamentally important phenomenon that has largely been overlooked, which may be a critical factor in AD pathogenesis.

## Results

### Determination of the critical micelle concentrations (CMCs) of saturated NEFAs

**Figure 1:**
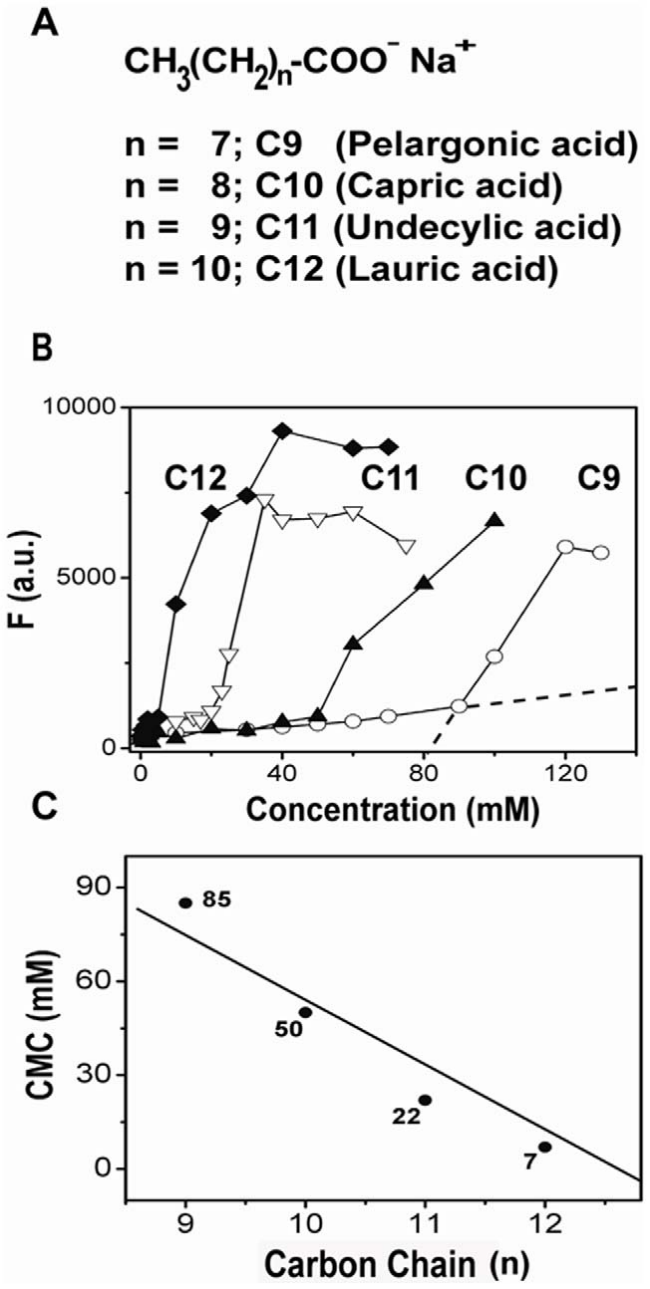
Critical micelle concentration (CMC) measurements of NEFAs used in this study. A) NEFAs used in this study: ‘n’ represents the carbon chain length, B) N-phenyl-1- naphthylamine (NPN) fluorescence response curves for fatty acids used in this study; C9 (○), C10 (▴), C11 (▿) & C12 (♦). Representative dotted lines for C9 shows the linear extrapolations of the curve to calculate CMC, which is the point of intersection ; C) CMC of fatty acids determined by these measurements plotted as a function of carbon chain length.

In this study, we have chosen medium-chain saturated NEFAs of varying carbon lengths including C9 C10, C11, and C12 (Figure 1A) to examine their effects on Aβ42 aggregation. Phase transitions of many surfactants including fatty acids have been widely investigated in the past and it is well known that sodium salts of NEFAs exhibit by and large a two phase behavior; above and below their CMCs [39]–[43]. However, more complicated phase behavior was observed as a function of ionization state of NEFAs and pH [44]. Nevertheless, at a given set of pH and buffer conditions, the presence of the two broadly-defined micellar and non-micellar phases have been well documented previously [39]–[43]. In order to determine the respective CMCs of NEFAs in the buffer (10 mM Tris-HCl, 50 mM NaCl, pH 8.0) used in the study, we used N-phenyl-1-naphthylamine (NPN) as a probe that fluoresces upon binding to micelles, as reported earlier [40], [45]. Figure 1B shows the normalized fluorescence titration curves for varying concentrations of NEFAs. The first ‘inflection point’ (indicated by extrapolated dotted lines for C9 in Figure 1B) that occurs upon increasing the fatty acid concentration was considered to be the CMC. Figure 1C shows the plot of CMCs determined from Figure 1B as a function of increasing carbon chain length. The estimates of CMCs obtained were in agreement with previous reports [40], [41]. The presence of micelles above the CMCs was also confirmed by dynamic light scattering analyses, whose size varied with carbon chain length (data not shown).

### Effect of saturated NEFAs on Aβ42 aggregation

**Figure 2:**
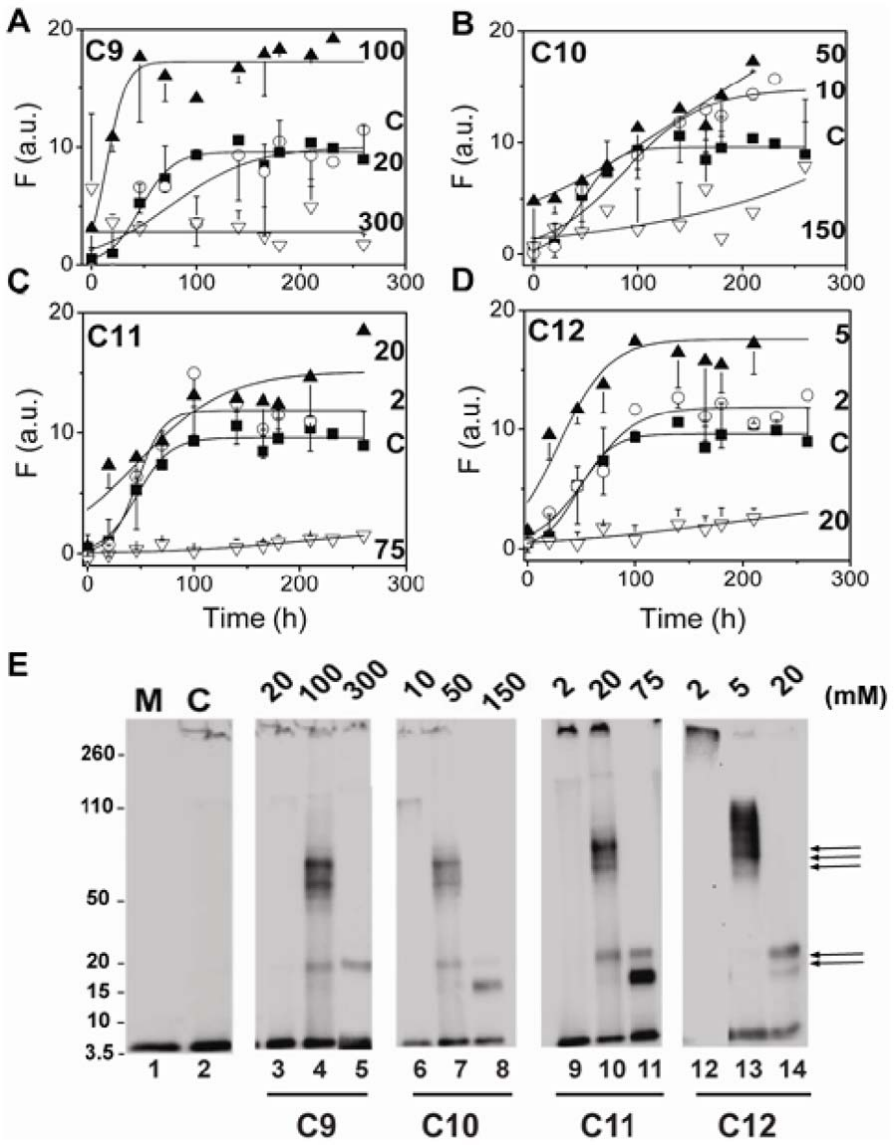
Dependence of Aβ42 aggregation on fatty acid concentration monitored by ThT fluorescence. Monomeric Aβ42 (25 μM) was incubated at 37°C in buffer alone (C; ▪) or with varying concentrations of fatty acids C9 (A), C10 (B), C11 (C) and C12 (D), above (▿), below (○) or near (▴) their respective CMCs. The numbers indicated inside the boxes are respective concentrations of fatty acids in mM units. The data are averages of three experiments with different Aβ42 purification batches. The data were fit with Eq 1; E) Western blots of the samples in A–D probed using monoclonal antibody, Ab9 after 128 h of incubation. The samples were run on a 12% bis-Tris acrylamide gels with Laemmli buffer. Double arrow indicates 4–5 mers while the triple arrow indicates 12–18 mers of Aβ42. ‘M’ represents the monomer control while ‘C’ represents Aβ42 sample in the absence of fatty acids. The numbers on top of the gels are fatty acid concentrations in mM units.

The aggregation kinetics of Aβ42 in the presence of NEFAs of varying carbon chain lengths, C9, C10, C11 and C12, was monitored by ThT fluorescence as shown in Figure 2A–D. To assess the effect of CMC of fatty acids on Aβ42 aggregation, based on previous reports on Aβ interactions with SDS and other surfactants [30] we chose three specific concentrations of fatty acids: well above (∼3–5 fold), well below (∼3–5 fold) and near the CMC. Incubation of 25 µM buffered (10 mM Tris, 50 mM NaCl, pH 8.0) Aβ42 alone at 37°C showed a typical sigmoidal pattern with a lag-time of∼57 h (Figure 2A–D, control) indicating the growth of fibrils. However, upon incubation with varying concentrations of fatty acids, three different types of aggregation behavior were observed. For concentrations of fatty acids well below their CMC, the rate of Aβ42 aggregation is similar to that of the control. For concentrations of fatty acids near their CMC, the rate of aggregation is significantly accelerated, and for concentrations well above the CMC aggregation is inhibited. This is clearly seen in Figure 2A for the C9 solutions, where concentrations of C9 near the CMC (100 mM) augmented the rate of Aβ42 aggregation with no observable lag-time, while in concentrations well above CMC (300 mM), the co-incubated sample failed to show significant ThT fluorescence over the 10-day incubation period. Incubations with concentration below the C9 CMC (20 mM), did not show any significant difference from the control sample in the absence of fatty acids. We observed that this phenomenon of increase and decrease in Aβ aggregation rates at near and well above the CMC respectively, is conserved for all fatty acids that were analyzed. Increased rates of Aβ42 aggregation were observed in incubations with 50 mM C10, 20 mM C11 and 5 mM C12 (▴ in Figures 2B, C and D respectively) concentrations that are near their respective CMCs. Similarly, Aβ42 incubations with concentrations above the CMCs (150 mM C10, 75 mM C11 and 20 mM C12) resulted in very low ThT fluorescence levels suggesting lack of aggregation (▿ in Figure 2B, C and D). Furthermore, the concentrations of fatty acids well below their CMCs did not have any significant effect on Aβ42 aggregation for all fatty acids observed (○ in Figure 2B, C and D). Appropriate blanks of ThT as well as bis-ANS fluorescent probes in 20 mM C12 fatty acid alone did not show significant increase in fluorescence intensity suggesting that the micelles do not interact with the probes to contribute significantly to the intensity observed.

Since the interfaces generated by micellar SDS have been previously observed to generate specific oligomeric Aβ42 species [30], we wanted to establish whether similar oligomeric forms are generated by NEFAs also. Therefore, aliquots of the samples after 130 h of incubation from the reactions in Figure 2A–D were subjected to SDS-PAGE analysis and immunoblotting using Ab9 monoclonal antibody as shown in Figure 2E. All samples were subjected to electrophoresis on a 12% bis-Tris acrylamide gels with Laemmli buffer. In an effort to resolve potential dimeric band that may be present, we attempted several runs on 4–12% NuPage® gels (Invitroen Inc). However, the presence of high concentrations of NEFAs (particularly in C9, C10 and C11 cases) obscured the banding patterns (data not shown). Nevertheless, in 12% gels, the control Aβ42 in the absence of fatty acid showed a monomeric band along with one that failed to enter the gel (band on the top of the gel), indicating the formation of fibrils as also observed by ThT fluorescence (Figure 2E, lane 2). Fatty acid concentrations well below the CMC that did not have significant effect on aggregation as observed by ThT fluorescence indicated a banding pattern that was similar to the control sample (Figure 2E; lanes 3, 6, 9 & 12). In sharp contrast, solutions incubated with fatty acid concentration near and well above CMC showed differences in the banding patterns. At fatty acid concentrations near their respective CMCs where aggregation rates were augmented, the samples showed a predominant 50–80 kDa band that corresponded to 12–18 mers of Aβ42 (lanes 4, 7, 10 & 13; indicated by triple arrows). In addition, a faint band at 20 kDa (5 mer; indicated by double arrows) along with fibrillar (F) and monomeric bands were observed (Figure 2E). Monomeric bands were observed in almost all samples, and it is likely that this is due in part to partial dissociation of higher molecular weight aggregates upon exposure to high concentrations of SDS during electrophoresis. Similarly, it is also reasonable to hypothesize that the faint 4–5 mer bands seen along with 12–18 mer bands may arise from partial dissociation of these oligomers. Nevertheless, at concentrations well above their respective CMCs where samples failed to show significant ThT levels, the presence of fatty acids resulted in a predominant 16–20 kDa band corresponding to 4–5 mers of Aβ42, in addition to a monomeric band (lanes 5, 8, 11 & 14). Similar band patterns were observed at approximately 220 h of incubation also (data not shown). Overall the data indicate that fatty acid concentrations above and near the CMC lead to show maximum differences in the aggregation rates and generate predominantly two distinct oligomeric species of Aβ42: 12–18 mers and 4–5 mers.

### Secondary structure changes during aggregation suggest subtle secondary structure differences between 12–18 mers and 4–5 mers

**Figure 3:**
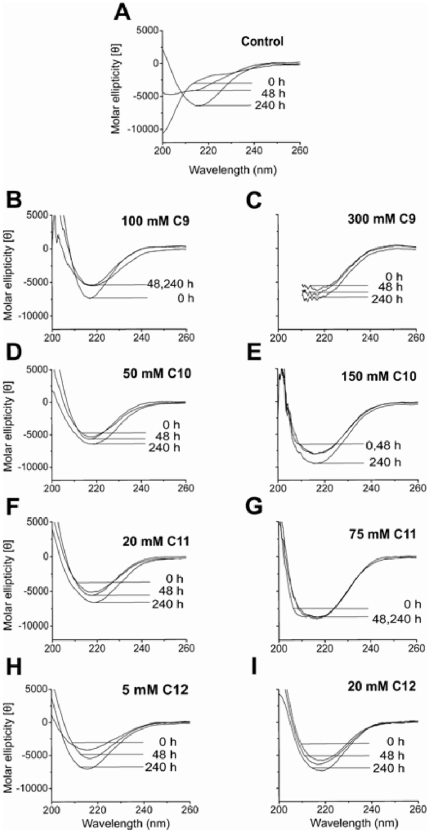
Secondary structure change indicated by far-UV CD during incubation of Aβ42 with varying concentrations of NEFAs, C9, C10, C11 and C12 near (left panel) or above (right panel) their respective CMCs.

In order to gain insights into the secondary structure changes in Aβ42 that occur during aggregation in the presence of different concentrations of fatty acids, we analyzed the samples using far-UV CD spectroscopy. Aliquots of samples incubated under identical conditions as those shown in Figure 2 were analyzed and data were collected using a CD spectropolarimeter every day for 10 days. The spectra for three specific time points, 0, 48 and 240 h, are shown in Figure 3. As expected, the control Aβ42 in the absence of fatty acids displayed a random coil conformation at 0 h that converted to a β-sheet over a 10-day incubation period with a negative minimum at approximately 216 nm, suggesting conversion into fibrils (Figure 3A). For the samples incubated with fatty acids, based on ThT and immunoblot results, we chose concentrations, near and above the respective CMCs, to examine by CD. All Aβ42 solutions containing the fatty acids, C9–C12, at both near- and above-CMC concentrations showed immediate conformational change from a random coil monomer. The resulting Aβ42/NEFAs CD spectra exhibited similar general features, with a minimum at approximately 216 nm, indicative of a predominantly β-sheet conformation (Figure 3B-I). The sharpness and amplitude of that feature, differed only subtly for near- and above-CMC NEFA conditions. This may be attributed to differences in relative contributions of the parallel and antiparallel β-sheets, and potential contributions from turn conformations, for example β-turn type I [46]–[48]. Incubations at NEFA concentration below CMC resulted in similar behavior to control samples with the conversion of random coil to β-sheet over the incubation period (Figure S1).

Unfortunately, data could not be collected below λ = 210 nm for the 300 mM C9 solution as the high concentration of the fatty acid resulted in prohibitively high absorbance of the sample. Collectively, the CD data suggest that both the 12–18 mers and the 4–5 mers are predominantly β-sheet, but they appear to contain subtle differences in their secondary structures. It also appears that there is an overall shift towards higher β-sheet structure for Aβ42 samples in the presence of NEFAs above the CMC as compared to the ones with near CMC. Based on this difference one may speculate that the contributions from conformational change in monomeric Aβ might contribute to the spectra in addition to the relative populations of 4–5 mer/12–18 mers. It is also noteworthy that β-sheet structures formed by 4–5 mers do not exhibit ThT fluorescence, unlike those formed by 12–18 mers.

### Oligomeric 12–18 mers and 4–5 mers differ in their morphologies

**Figure 4:**
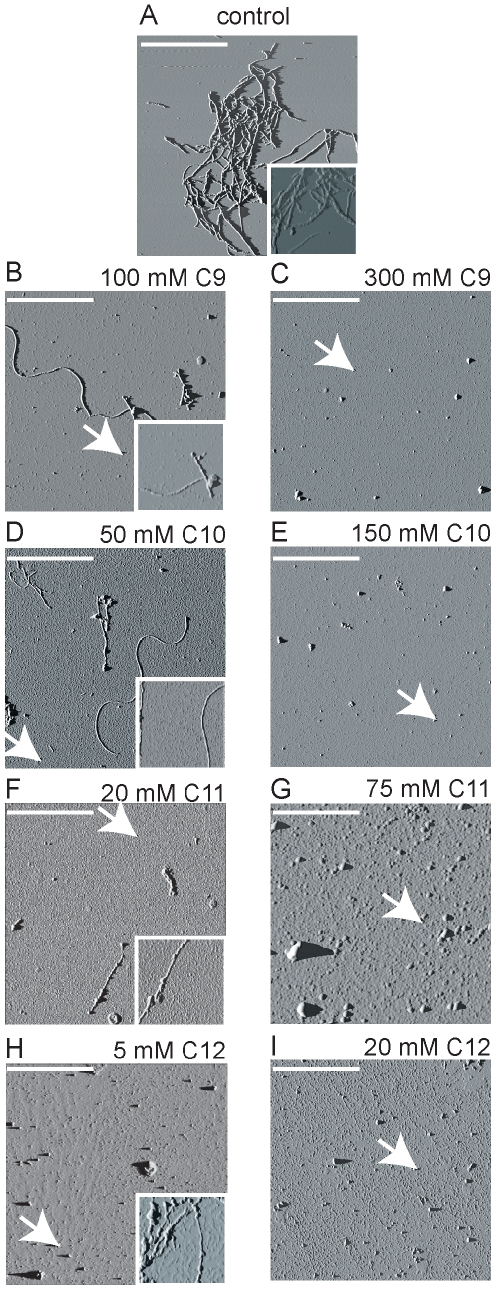
Aliquots of the samples from **Figure 2** were probed to see the morphologies of Aβ42 aggregates generated in the presence of varying concentrations of fatty acids either near or above the CMC by atomic force microscopy (AFM). A) control ; B&C) 100 & 300 mM C9; D & E) 50 & 150 mM C10; F & G) 20 & 75 mM C11; H & I) 5 & 20 mM C12. Amplitude images with scale of 0–0.4 volts are presented. The white arrows indicate typical oligomeric samples. The white scale bar represents 1 µm and each square represents 5×5 µm field and the inset shows a different field of dimensions 1×1 µm.

The morphologies of the structures generated in solutions with different concentrations of fatty acids were examined by AFM. Aliquots of samples taken after 240 h of incubation from Aβ42 solutions containing fatty acid concentrations near and above the CMC were deposited on freshly cleaved mica surfaces as described in the experimental section, dried and imaged in AFM tapping mode (Figure 4). Clear differences in the morphologies of the samples taken from the different fatty acid solution concentrations were observed. The control Aβ42 sample showed clusters of fibrillar material across the entire mica surface (Figure 4A). Images of samples taken from solutions with fatty acid concentration near the CMC exhibited mixtures of fibrillar and smaller rounded/oblong shaped features dispersed across the mica surface (Figures 4 B,D,F,H), while those taken from solutions above CMC revealed only rounded/oblong features (Figures 4 C,E,G,I). The AFM analysis corresponds well with the results obtained from SDS-PAGE, where it was shown that the fatty acid solutions near CMC generated predominantly 12–18 mers and fibrils, with smaller amounts of 4–5 mers, while those above CMC generated 4–5 mers and no fibrils (Figure 2E). Analysis of the height profiles of the AFM surfaces revealed that the majority of the features had cross-sectional heights of 6–8 nm. Measured average cross-sectional height of the fibrils in the control sample was 6.1±0.8 nm. The small rounded/oblong features in the samples prepared from near CMC fatty acid concentration showed average height of 7.9±2.9, while the fibrillar features showed average height of 5.6±1.3 (Figure 4 B,D,F,H). The oblong features in the samples produced from high concentration of fatty acid had average height of 6.7±1.0 nm (Figure 4 C,E,G,I). The small percentage of large, irregular globular features observed in some of the samples presented heights ranging from 12 to 20 nm. We attribute these larger globules to aggregates of oligomers. Thus, the heights of the features are generally similar in all samples, but the aspect ratio varies with NEFA concentration. For example, fibrils, circular structures and short elongated structures are clearly seen in Figure 4 D. No such elongated or fibrillar structures are visible in solutions with high fatty acid concentrations. Collectively, the AFM data suggest that the structures generated by near CMC concentrations (predominantly 12–18 mers with some fibrils) are morphologically different from those generated in high concentrations of fatty acids (predominantly 4–5 mers).

### Seeding experiments suggest 12–18 mers and 4–5 mers may have structural dissimilarities

Results from ThT fluorescence, immunoblotting, CD spectroscopy and AFM experiments suggested that 12–18 mers and 4–5 mers may have fundamentally different structures and morphologies. It is well known that the Aβ aggregation is a nucleation dependent process and that the lag phase of aggregation can be eliminated by adding preformed aggregates as ‘seeds’ to monomeric Aβ42 [49]. The structure and morphology of the seeded aggregates as well as the efficiency of seeding, which directly reflects the elongation rates, depends on the structure of the ‘seed’ itself [50]. In other words, if the oligomers formed are structurally compatible with the emerging structure of fibrils, they will seed the on-pathway fibril formation process. Thus, the seeding process is one way of evaluating the structural assembly of oligomers and consequently pathways of aggregation as reported previously [50]–[54].

**Figure 5:**
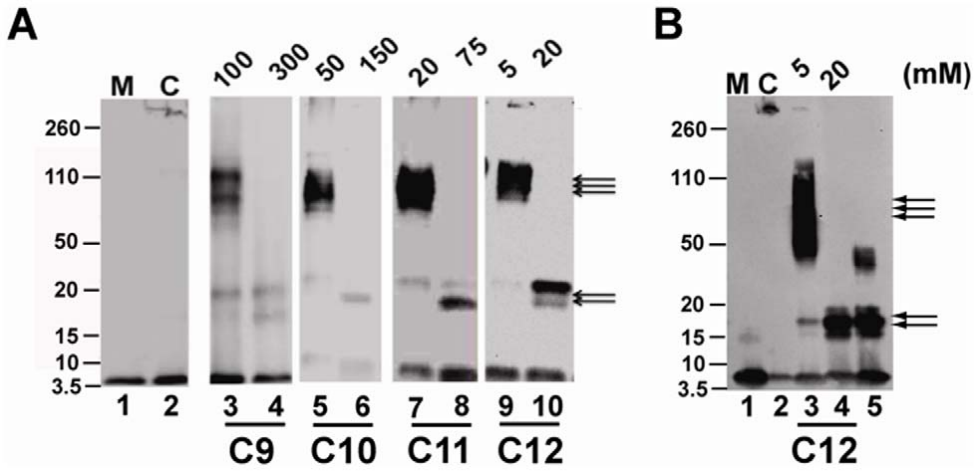
Western blots of Aβ42 samples after 48 h of incubation in the presence of fatty acids near and above the CMC, respectively. A) SDS-PAGE was run on a 12% bis-tris acrylamide gels in Laemmli buffer. Double arrow indicates 4–5 mers while the triple arrow indicates 12–18 mers. The monomeric Aβ42 and control (in the absence of fatty acids) are represented by M and C respectively. B) Western blots of Aβ42 samples incubated in the presence of C12 NEFA near and above the CMC run on 4–12% NuPage gels for comparison. M and C represent the monomeric, and control Aβ42 samples (lanes 1 and 2). Lanes 3 and 4 are C12 incubations with 5 and 20 mM respectively while lane 5 is the incubation of Aβ42 in 2 mM SDS as a positive control.

**Figure 6:**
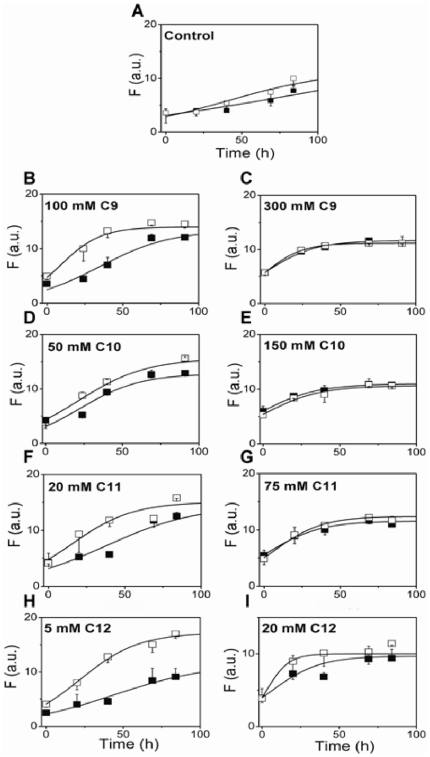
Seeding experiments with oligomers. (A–I) Aβ monomer (25 µM) was incubated alone or with 10% (m/v) seed of 48 h incubated samples of Figure 5 at 37°C and monitored by ThT fluorescence. The figures on the left and right panel represent concentration near and above the CMCs respectively. The unseeded control (▪) is same reaction as the seeded (□) one, but lacks seed.

For this experiment, we incubated ‘seed-free’ monomeric Aβ42 (purified by SEC, see Experimental Procedures), with fatty acids (C9–C12) at concentrations near and above their respective CMCs under similar conditions as those described for Figure 2. After 48 h of incubation aliquots of the samples were electrophoresed and immunoblotted to evaluate the formation of oligomeric species. As expected, the samples indicated the presence of 12–18 mers in concentrations near, and 4–5 mers at concentrations above the CMC (Figure 5A) in 12% acrylamide gels. In order to ensure there are no other bands in between monomer and 4–5 mers, we electrophoresed Aβ42 samples incubated in C12 on a 4–12% NuPage gels (lanes 3 and 4, Figure 5B). We have also included similar Aβ42 incubation in 2 mM SDS as a positive control (lane 5, Figure 5B) [30]. We did not see any discrete bands in between monomeric and 4–5 mer bands in any of the samples. In parallel, 10% (v/v) aliquots of the samples were used as ‘seeds’ by adding them to a freshly purified Aβ42 monomeric sample solution. This ‘seeded’ reaction was incubated at 37°C and the aggregation was monitored by ThT fluorescence as mentioned previously (Figure 6). Samples of 10% solutions containing fatty acid alone without Aβ were used as controls. The control Aβ42 (aggregated in the absence of fatty acids) was able to seed with marginal efficiency as seen by a slight increase in the rates of aggregation for the seeded sample (Figure 6A). This was expected, as control Aβ42 might not have generated enough oligomers that can act as seeds within 48 h of incubation. Seeds containing 12–18 mers generated in the presence of fatty acids at concentrations near their CMCs were clearly able to augment the rates of Aβ42 aggregation compared to the control (Figure 6 B, D, F & H). Among them, 100 mM C9 and 5 mM C12 showed a dramatic increase in aggregation rates in comparison to 50 mM C10 and 20 mM C11 (Figure 6 B, D, F & H). However, the seeds containing 4–5 mers formed in high concentrations of fatty acids clearly failed to show any effect on Aβ aggregation rates suggesting inability of these oligomers to seed fibril formation (Figure 6C, E, G & I). These results suggest that the 12–18 mers may be structurally similar to fibrils and hence, may be intermediates of the fibril formation ‘on-pathway’. The 4–5 mers seem to be structurally dissimilar to 12–18 mers and may represent the intermediates of an alternative, ‘off-pathway’. The inability of these aggregates to convert to fibrils in high fatty acid concentrations as observed in Figure 2A further supports this conclusion.

### Oligomeric 4–5 mers and 12–18 mers differ in their thermodynamic stabilities

**Figure 7:**
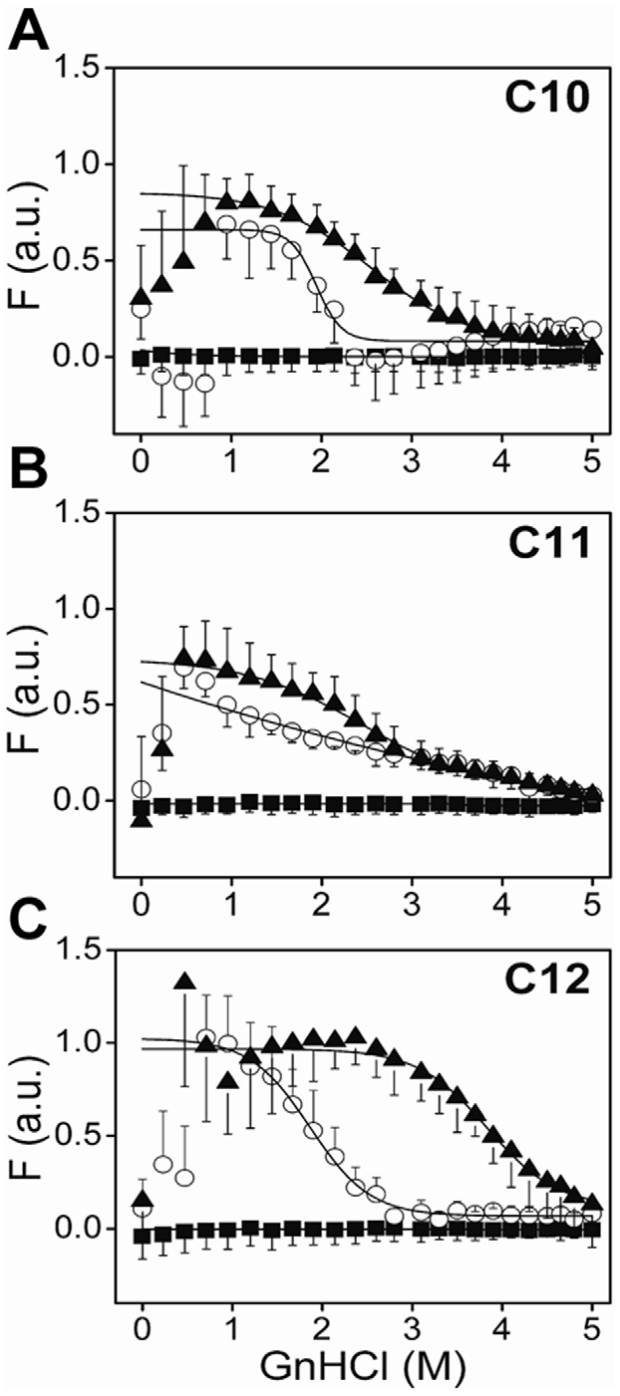
Thermodynamic stabilities of the oligomers determined by GnHCl denaturation experiments. Aβ42 (25 µM) was incubated alone (▪) or with NEFAs C10 (A), C11 (B) and C12 (C)under similar conditions as those represented in Figure 2, at concentrations near (○) and above (▴) the CMCs of the fatty acids. The samples after 48 h incubations were subjected to denaturation by the addition of 6M stock of GnHCl solution. The reactions were monitored by tyrosine intrinsic fluorescence. Three scans were averaged and the areas under the peaks normalized and plotted against GnHCl concentrations. The data was fit using Boltzmann’s sigmoidal fit (Eq 2) by Origin 7.0.

Oligomeric Aβ42 4–5 mers and 12–18 mers clearly showed a difference in their ability to seed Aβ42 fibril formation, which is likely a consequence of dissimilarity in their structural assembly. If it is so, we reasoned that the two species may also differ in their thermodynamic stabilities. To assess how 4–5 mers and 12–18 mers differ in their equilibrium stabilities, we observed the temporal denaturation melting curves of the Aβ incubations with fatty acids using GnHCl as a denaturant. We titrated the co-incubated samples containing 4–5 mers and 12–18 mers with increasing concentrations of GnHCl and monitored the changes in intrinsic tyrosine fluorescence as shown in Figure 7. The monomeric Aβ42 control did not display melting changes, as is expected from a natively unstructured protein (▪, Figure 7A, B & C). However, aggregates formed in the presence of fatty acids at concentrations both near (12–18 mer), and above their CMCs (4–5 mer) showed significant fluorescence intensity that decreases to Aβ42 control levels with increasing GnHCl concentrations (○ & ▴; Figure 7). The first four or five data points collected at low GnHCl concentrations were erratic, especially in high concentrations of fatty acids. We think this is probably due to the slight precipitation of fatty acids that occurs upon the addition of GnHCl. However, at higher loadings of GnHCl, the solutions appear to stabilize. For the same reason, it was not possible to collect reliable data in C9 solutions, and hence this is not included. For the other NEFA solutions, the first five data points were not included in our curve fittings. It is evident from the data that for all three fatty acids (C10, C11 and C12), 12–18 mers ‘melt’ earlier than the 4–5 mers (Figure 7). The apparent melting denaturant concentration (*C_M_*) values for the 12–18 mers (○; Figure 7) corresponded to 1.94±0.05, 0.87±0.1 and 1.86±0.04 M for C10, C11 and C12 respectively. The samples containing 4–5 mers (▴; Figure 7), on the other hand, required higher concentrations of GnHCl to denature the oligomers, with *C_M_* values corresponding to 2.55±0.03, 2.38±0.06 and 3.83±0.09 M for C10, C11 and C12 respectively. The apparent melting of 4–5 mers formed at higher concentrations of NEFAs may also be attributed to the ‘shielding’ effect of oligomers by NEFAs, which may not be indicative of their inherent stability. In order to rule out this possibility, we monitored similar GnHCl melts of a protein that is unrelated to Aβ, called, human granulin A (hGRN-A) as a negative control. hGRN is a 7 kDa protein (comparable in size to Aβ) involved in tumorigenesis and is not known to aggregate [55]. Upon incubation with 5 and 20 mM C12 for 48 h, melts were preformed similar to those in Figure 7 (Figure S2). We observed that both 5 and 20 mM C12 resulted in comparable melting concentrations of GnHCl with only a marginal shielding effect observed for 20 mM C12. This difference in melting concentrations is much smaller than the one between 5 and 20 mM C12 incubation with Aβ42 (Figure 7C) indicating thermodynamic stability of the aggregates contributed to the difference in large part and not just the shielding effect of NEFAs. It was interesting to observe that 4–5 mers formed in C12 appeared to be more stable than the rest of the 4–5 mers. The denaturation experiments collectively suggested that the 4–5 mers were more stable than 12–18 mers, further complementing the other data that indicated the two oligomeric species may be structurally different.

### Oligomeric 12–18 mers can be isolated

**Figure 8:**
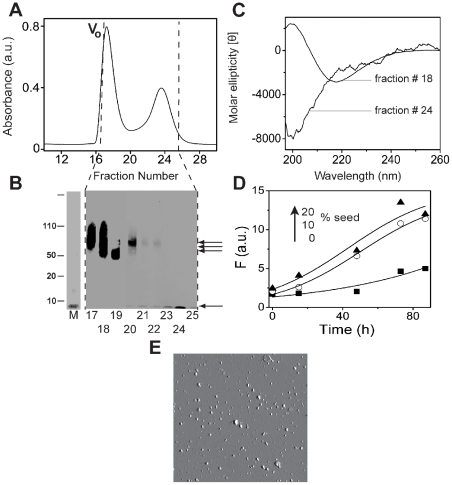
Oligomeric 12–18 mers can be isolated and characterized. Aβ42 (50 mM) was incubated with 5 mM C12 for 48 h at 37 °C to generate 12–18 mers. A) The sample was fractionated on a Superdex-75 SEC with a flow rate of 0.5 ml/min, V_o_ indicates void volume, B) Immunoblots of the fractions, developed with Ab9 antibody indicates the separation of 12–18 mers and monomers, C) Far-UV CD spectra of the fractionated samples, fraction 18 and 24 that show β-sheet and random coil structures respectively. and, D) seeding experiment was performed with 10% (○) and 20% (▴) of fraction 18 as seeds with 25 µM Aβ42 monomer at 37°C Control in the absence of seeds is shown as (▪) and the data was fit with Eq 1 as in Figure 2. E) AFM image (5×5 µm) of fraction 18 (12–18 mer) shows largely a bimodal distribution of spherical particles.

Next, we wanted to evaluate whether we could isolate 4–5 mers and 12–18 mers and remove monomers, fibrils and possibly the fatty acids that may be present along with them. This will not only facilitate the exploration of the molecular features of the oligomers, but also will determine whether the oligomers are stable in the absence of fatty acids. To do so, we incubated 50 µM Aβ42 monomers in 5 and 20 mM C12 for 48 h to generate 4–5 mers and 12–18 mers as shown previously in Figure 5. The 5 mM C12 samples that generated 12–18 mers were then subjected to fractionation by SEC in Superdex-75 column as shown in Figure 8A. The sample fractionated into void volume and inclusion volume peaks. The fractions were then subjected to electrophoresis and immunoblotting (Figure 8B), which indicated the presence of 12–18 mers exclusively in the void volume peaks (fractions 17–18) as well as in the partially included volume (fractions 19–20), and monomers in the inclusion volume (fractions 23–25). The data clearly suggested that the 12–18 mers could be fractionated in a fairly homogeneous form. We checked the structural integrity of the isolated 12–18 mer by far-UV CD (Figure 8C), which showed a well-defined β-sheet structure for the sample in fraction 18 as compared to the random-coil for the fractionated monomer in fraction 24. In order to determine whether the isolated 12–18 mers were able to ‘seed’ Aβ fibril formation, we performed a ‘seeding’ experiment as described previously. As Figure 8D shows, 10 and 20% (molar) seeds of the isolated 12–18 mers were able to increase the rate of Aβ aggregation as observed for the oligomers prior to fractionation. AFM images of the isolated 12–18 mers showed spherical structures on the mica surface with a bimodal distribution of sizes; one with average height 9.1 nm±1.7 nm and a second of 19.3 nm±1.0 nm (Figure 8E). Quantitation of any residual NEFA present in the isolated 12–18 mers showed only<0.5% present (data not shown). These result suggested that the isolated 12–18 mers maintained their structural integrity after being subjected to SEC. Similar attempts to isolate 4–5 mers in a similar fashion were not successful (data not shown). We are currently in the process of addressing this issue along with analyzing the structural assembly of 12–18 mers that will be published later.

## Discussion

Interfaces are known to have profound effect on Aβ aggregation. The data presented here provide some unique insights into the phenomenon of interfacial aggregation in the presence of medium-chain saturated NEFAs. The data show that different concentrations of fatty acids seem to induce multiple pathways of Aβ42 aggregation. More importantly, the aggregation pathways adopted by Aβ appear to be dependent on the nature of the interface (generated by the specific concentration of NEFAs), and not the type of fatty acid used. For example, similar 12–18 mers or 4–5 mers are generated near and above the CMC of the fatty acid irrespective of its carbon chain length. In addition, increasing Aβ: NEFA ratios resulted in a proportional increase in fibrils for those incubated∼CMC, where 12–18 mers are formed (Figure S3). However, the amount of 12–18 mers seem to remain stable. Similarly, increase in Aβ:NEFA ratio for incubations>CMC (4–5 mers) resulted in the formation of 12–18 mers (Figure S2) suggesting ratios of Aβ and NEFA are important in dictating pathways as well as the nature of oligomers formed. Furthermore, the nature of the interface (micellar or otherwise) and not just the ratio of Aβ: fatty acid seems to play a major role in dictating pathways. This is evident from the aggregation patterns of Aβ42 in the presence of 20 mM C9, C11 and C12, at constant Aβ: fatty acid ratios (Figure 2). However, the concentration of 20 mM fatty acid falls well below the CMC for C9 and near the CMC for C11, while the same concentration falls well above the CMC for C12 (Figure 1). Clearly, at this concentration, C9 does not have any effect of Aβ42 aggregation while C11 and C12 induce the formation of 12–18 mers and 4–5 mers respectively (Figure 2E), indicative of the importance of CMC in Aβ interactions.

### Oligomeric 12–18 mers and 4–5 mers are ‘off-pathway’ products with subtle structural differences

**Figure 9:**
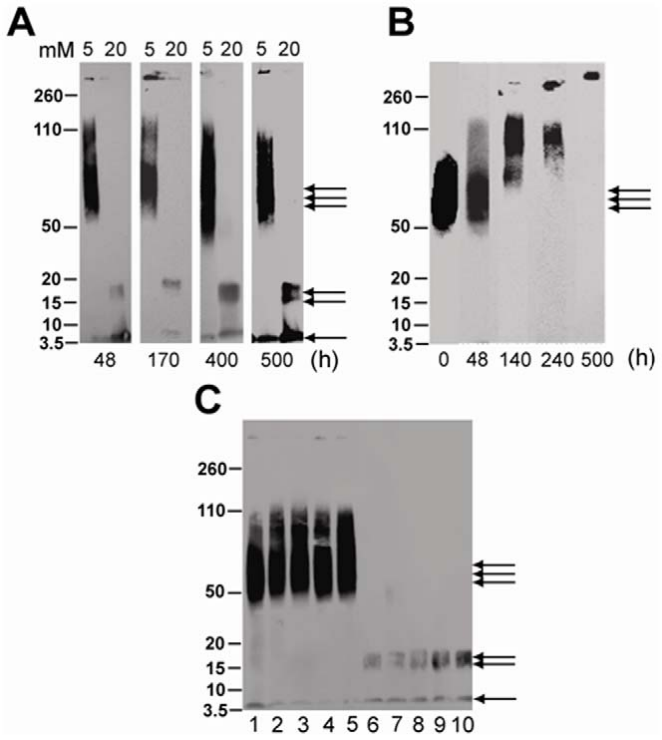
Oligomeric 12–18 mers as well as 4–5 mers are formed along ‘off-pathway’. All samples were electrophoresed on 4–12 % NuPage gels with MES running buffer. A) Immunoblots of incubations of buffered 25 µM Aβ42 with 5 and 20 mM C12 for∼500 h at 37°C similar to Figure 2. Double and triple arrowheads indicate 4–5 mers and 12–18 mers respectively. B) Immunoblots of fractionated 12–18 mers (9 µM) of Aβ42 by SEC (Figure 8) in 20 mM TrisHCl, 50 mM NaCl, pH 8.0 incubated at 37°C at the indicated times. C) Effect of fibril inhibitors like Congo red (CR) & Rifampicin (Rfn) on Aβ42 oligomer formation. Lanes 1–5 show 25 µM Aβ42 incubations with 5 mM C12: lane 1- with no inhibitor; lanes 2 and 3- with 6- and 8-fold molar excess of CR; lanes 4 and 5- with 6- and 8-fold molar excess of Rfn. Lanes 6–10 show similar Aβ42 incubations with 20 mM C12: lane 6- with no inhibitor; lanes 7 and 8- with 6- and 8-fold molar excess of CR; lanes 9 and 10- with 6- and 8-fold molar excess of Rfn.

At concentrations near the CMC, the fatty acids were able to induce the formation of Aβ42 12–18 mers within the first 24–48 h of incubation that were present during the rest of the 10-day incubation period. The samples containing 12–18 mers also displayed a ‘ThT-positive’ character and showed increased rates of aggregation. It was difficult to establish whether the oligomeric species themselves bound ThT or the presence of some fibrils in these samples (Figure 4) was responsible for increased ThT fluorescence. Nevertheless, it seems probable that the 12–18 mers are formed in the same pathway Aβ fibril formation. Evidence in support of this comes from seeding experiments in which both non-isolated and isolated 12–18 mers were able to accelerate Aβ fibril formation (Figures 5 and 7). However, two separate experiments suggest 12–18 mers are formed as ‘off-pathway’ products; First, the incubations for longer periods of time (∼500 h) failed to result in the complete depletion of the 12–18 mer bands as expected for on-pathway intermediates (Figure 9). Secondly, known inhibitors of the ‘on-pathway’ fibril formation such as as Rifampicin and Congo Red [33], [56], [57], failed to inhibit the formation of 12–18 mers (Figure 9). Although seeding is suggestive of intermediates being ‘on-pathway’, the ability of 12–18 mers to seed fibril formation may indicate their structural similarity with the on-pathway fibrils. Interestingly, isolated 12–18 mers free of monomers and NEFAs slowly converted to fibrils over 500 h of incubation at 37°C (Figure 9). Together, the data suggests that the 12–18 mers are trapped as ‘off-pathway’ intermediates, stabilized by NEFAs. However, once NEFAs are removed (isolated 12–18 mers), they are able associate with each other to form larger aggregates and to eventually fibrils (Figure 9). The rate of fibril formation is however slow, suggesting that 12–18 mers may be kinetically trapped as ‘off-pathway’ species.

The 4–5 mers on the other hand, clearly behaved as ‘off-pathway’ products. For the entire incubation period, samples containing predominantly 4–5 mers (in high fatty acid concentrations) did not show increase in ThT fluorescence levels. Similar to 12–18 mers, they also failed to convert to fibrils over long incubation periods∼500 h (Figure 9). In addition, 4–5 mer-containing samples were fairly homogenous without fibrils or any higher molecular weight species (Figure 2E and 4). Furthermore, 4–5 mers could not ‘seed’ Aβ42 fibril formation, supporting the suggestion of their structural incompatibility to nucleate on-pathway aggregation. Unfortunately, it was not possible to fractionate 4–5 mers over an SEC column, as was done for 12–18 mers, to test their seeding ability and we are currently in the process of addressing this issue. Nevertheless, the collective data suggest that 12–18 mers and 4–5 mers are oligomers that are trapped (at least transiently) in a local energy minimum, or in other words, ‘off-pathway’. It is also likely that the fatty acids stabilize the oligomer structure in such way that they are unable to aggregate and that the removal of fatty acid may destabilize the oligomer. But 12–18 mers and 4–5 mers exhibit only subtle structural differences (Figure 3, CD spectra) that are large enough to be differentiated by ThT binding. We are currently investigating these aspects and will be published later.

**Figure 10:**
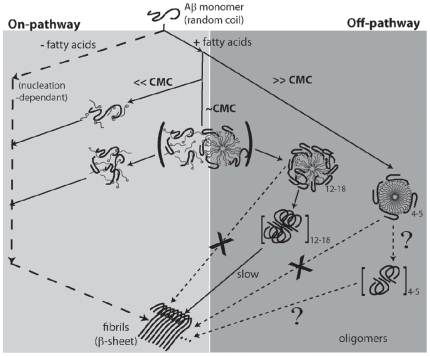
Schematic model of Aβ42 aggregation pathways in the presence of NEFAs based on the data obtained in this study. The square brackets indicate isolated oligomers while the question mark indicates that these parts were not explored in this study.

The precise nature of Aβ-NEFA interactions is not known. However, one can speculate the reasons for the observed Aβ42 aggregation behavior based on our results and generate a model as shown in Figure 10. Assuming that the non-micelle ← → micelle transition is a concerted reaction with an equilibrium constant, K_D_ being equal to the CMC value, it is clear that the interaction of Aβ with fatty acids well above their CMCs is exclusively with the micellar form (Figure 10). The anionic interface provided by the micelle seems to accommodate 4–5 monomers of Aβ42 that interact among themselves to form oligomeric 4–5 mers. Based on our initial thermodynamic analysis, these 4–5 mers are more stable (may be stabilized by the micelles themselves) than the 12–18 mers (Figure 7). Hence, it is possible that they are trapped in a local energy minimum as ‘off-pathway’ products. The non-micellar forms of NEFAs (<<CMC) do not seem to affect Aβ42 aggregation (Figure 2). However, at concentrations near the CMC, the dynamic equilibrium that exists during non-micelle ← → micelle transition seems to have unique effects on Aβ42 aggregation as discussed above. It is possible that due to this dynamic equilibrium, the interface formed is not well defined and a different mode of interaction exists between Aβ and the fatty acids as shown in Figure 10. This would also mean that the 12–18 mers may be kinetic intermediates along the ‘off pathway’ as opposed to the 4–5 mers, which are possibly thermodynamic in nature.

### Effects of NEFAs and SDS on Aβ42 aggregation are similar yet with subtle differences

The collective data suggest that the SDS-induced Aβ42 oligomer formation that was observed previously [30] can be recapitulated with high fidelity in NEFAs, and this supports our hypothesis that interfaces can dictate multiple Aβ aggregation pathways. However, there are some inherent differences between the fatty acid- and SDS-induced Aβ42 oligomers: (i) Aβ42 was able to form 2–4 mers at concentrations just below the CMC of SDS (2 mM), whereas with saturated C9–C12 NEFAs, 4–5 mers are formed only at concentrations well above their respective CMCs; at concentrations near the CMC, NEFAs increase the rates of Aβ aggregation and form 12–18 mers; and (ii) low concentrations of SDS (well below its CMC) increased the rates of Aβ aggregation while similar concentrations of NEFAs did not have any effect on Aβ42 oligomerization. These differences probably suggest that the significance of the surfactant anionic charge density in generating the interface which in turn affects Aβ aggregation pathways.

### All oligomeric species need not be intermediates of the fibril formation pathway: Physiological relevance

Several groups have focused attention on identifying and characterizing Aβ oligomers both *in vitro* and *in vivo*, which has led to several important findings. It was widely believed that these oligomers are intermediates along the fibril formation pathway (on-pathway). However, the polymorphism among Aβ aggregates suggests that there may be more than one pathway. Only a handful of reports indicate that the oligomers may not be obligatory intermediates to fibril formation but may populate alternate pathways from the classic nucleation-dependant one [23], [33], [58], [59]. It is important to understand the pathways of aggregation because if some oligomers are indeed formed as ‘off-pathway’ products, their half-life can be significantly longer than that of the ‘on-pathway’ oligomers, resulting in prolonged toxicity to neuronal cells. From the data presented here, we can hypothesize that some lipids and fatty acids may promote the formation of oligomers as ‘off-pathway’ products, and that the pathway may be dictated by the concentration and relative ratios of lipid to Aβ, which may be a significant aspect of Aβ amyloid biology.

The phenomenon of interfacial aggregation has been known for quite some time and many physiological interfaces are known to profoundly affect Aβ aggregation. Amphipathic Aβ peptide is known to have strong affinity for membranes that appear to affect the early stages of Aβ aggregation significantly [14], [18], [60]–[63]. Anionic phospholipids and GM1 ganglioside-containing lipid rafts are reported to increase the rates of Aβ aggregation [21], [22], [26], [61], [64], [65]. NMR determination of the interactions between Aβ40 and phosphatidylglycerols indicated that the interactions were largely restricted to the surface of lipids [66]. In addition to lipids, Barghorn and colleagues have reported that oligomers generated in fatty acids such as lauric acid, oleic acid and arachidonic acid were able to inhibit hippocampal LTP in Tg2576 mice [32], suggesting that they are toxic to the neuronal cells. The effect of physiological interfaces does not seem to be limited to Aβ alone. Many amyloidogenic proteins such as α-synuclein, β2-microglobulin, apolipoprotein C-II and prions are reported to have unique effects in the presence of interfaces [40], [45], [67]–[70]. Recently, a bacterially expressed recombinant prion protein, PrP converted to an infectious form, PrP^Sc^ that was shown to propagate only upon binding to anionic phosphatidyl membrane surfaces [71]. Given its physiological significance, lipid-induced interfacial aggregation can be pathologically significant in neurodegenerative diseases, especially with Aβ aggregation in AD. In the same context, the results discussed in this manuscript summarize an important phenomenon of Aβ amyloidogenesis, which is as follows: It is evident that interfaces can affect Aβ aggregation in more than one way, particularly in dictating multiple pathways. Given the mobility of cell membrane and lipid rafts in cellular environments that may result in concentration variations along the surface, it is possible that the ratio of Aβ and lipid components along with stoichiometry of interactions between the two may vary. This can potentially lead to the generation of ‘off-pathway’ oligomeric products, and since such products can have longer half-lives than ‘on-pathway’ intermediates, they may cause prolonged and extensive damage to the neuronal cells.

## Materials and Methods

### Materials

Aβ42 was synthesized at the Peptide Synthesis Facility at the Mayo Clinic (Rochester, MN) using routine Fmoc chemistry. MALDI-ToF mass spectrometry revealed>90% purity of both peptides. SDS, bovine serum albumin, and thioflavin T were procured from Sigma (St. Louis, MO). All fatty acids were purchased as sodium salts from NuCheck Prep Inc (Elysian, MN). All other chemicals were obtained from VWR Inc.

### Preparation of Aβ42 monomers

Lyophilized stocks of synthetic Aβ42 were stored at −20°C, desiccated. Prior to the experiments, any pre-formed aggregates that may have been present were removed via size exclusion chromatography (SEC) as previously reported[30]. Briefly, 2−3 mg of the peptide were dissolved in 0.5 ml of 30 mM NaOH, and allowed to stand at room temperature for 15 min before loading onto a Superdex-75 HR 10/30 size exclusion column (SEC) (GE Life Sciences) attached to an AKTA FPLC system (GE Healthcare, Buckinghamshire). The column was pre-equilibrated in 20 mM Tris-HCl (pH 8.0) at 25°C and was run at a flow rate of 0.5 mL/min. One minute fractions were collected. Concentrations of the purified fractions were estimated by UV-Vis spectroscopy on a Cary 50 spectrophotometer (Varian Inc) using a molar extinction coefficient (ε) of 1490 cm^−1^ M^−1^ (www.expasy.org) that corresponds to the single tyrosine residue within Aβ42. Peptide integrity after SEC was again confirmed by MALDI-TOF mass spectrometry that yielded a peak corresponding to a monoisotopic molecular mass of 4516.31 Da in a good agreement with a calculated mass of 4514.13 Da. Monomeric Aβ42 fractions were stored at 4°C and were used within three days of purification in all experiments to avoid the presence of pre-formed aggregation in our reactions.

### Aβ aggregation reactions

All reactions and measurements were made at room temperature unless otherwise noted. Reactions were initiated in siliconized eppendorf tubes by incubating appropriate concentrations of freshly purified Aβ42 monomer in buffer without agitation. Aggregation kinetic parameters were obtained by monitoring the reaction with thioflavin T (ThT) and fitting fluorescence data points to the sigmoidal curve in eq. 1[53] using Origin 7.0.



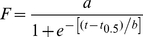
(Eq 1)


In this equation *t* is time, *a* and *b* are fixed parameters, and *t*_0.5_ is the time to reach half-maximal thioflavin-T fluorescence. Lag times were equal to *t_0.5_* − 2*b* for each fitted curve.

### Measurement of critical micelle concentration (CMC)

CMCs of fatty acids were determined using N-phenyl-1-naphthylamine (NPN) from Sigma (St. Louis, MO) as a fluorescent probe. Fluorescence measurements of NPN in the presence of fatty acids were acquired at the excitation wavelength of 340 nm and scanning emission wavelengths spanning 400–500 nm were monitored on a Cary Eclipse spectrometer (Varian Inc.). Fatty acid mixtures contained 0.5 mM–170 mM fatty acid along with 50 mM NaCl in 10 mM Tris buffer, pH 8.0. NPN was added to a total concentration of 1.5 µM and the solution was incubated for 30 minutes at 37°C. The data points were fitted to obtain a linear curve using Origin 7.0.

### Polyacrylamide gel electrophoreses (PAGE) and immunoblotting

In our experiments, we have used both 12% bis-Tris as well as 4–12% NuPage gels. Samples were dissolved in loading buffer (1 X Laemmli buffer for 12% bis-Tris gels; MES buffer for 4–12% gels) containing 1% SDS, applied without heating to 12% acrylamide gels containing bis-Tris, or 4–12% NuPage gels and resolved in Laemmli running buffer with 0.1% SDS. Dye-linked MW markers (Blue Plus2 Prestained Standards, Invitrogen) were run in parallel for calibration. Gels were electroblotted onto 0.45 µm Immobilon® nitrocellulose membranes (BioTrace™ NT, Life Sciences Inc). Blots were boiled in a microwave oven in PBS for 2 min and were blocked overnight with 1X PBS containing 5% nonfat dry milk and probed (3–5 h) with 1∶1000–1∶2500 dilutions of monoclonal antibodies Ab9, which detects amino acid residues of Aβ (1–16). Blots were then incubated with an anti-mouse horseradish peroxide (HRP) conjugate and developed with ECL reagent (Thermo Scientific).

### Fluorescence spectroscopy

ThT fluorescence (F) was monitored in a microcuvette with a Cary Eclipse spectrometer (Varian Inc) after 15-fold dilution of Aβ42 samples into 5 mM Tris-HCl (pH 8.0) containing 10 µM ThT. Continuous measurements of F were taken for 1min with the excitation and emission wavelengths fixed at 450 and 482 nm respectively, and the excitation and emission slits set at 10 nm. The fluorescence blanks were subtracted from the respective data and the average F value was determined.

### Atomic force microscopy (AFM)

Mica was cleaved using a razor blade and taped to a magnetic sample holder. The mica stub was then covered with a 3-aminopropyl-triethoxy silane (APTES) solution (500 µL APTES in 50 mL 1mM acetic acid) for 15 minutes. The APTES solution was then decanted and the mica was rinsed with 150 µL of deionized water, four times. After rinsing, the mica stub was dried with compressed N_2_ gas and stored in a desiccator for an hour. Next, 150 µL of 0.1–0.25 µM Aβ sample was added to the mica and allowed to adsorb for 20 minutes. The sample was then decanted and the mica stub was rinsed with 150 µL of deionized water, four times. Finally, the mica stub was dried with compressed N_2_ gas and stored in a desiccator until imaging. The surface topography of each sample was explored by imaging the peptide after it had been adsorbed onto APTES treated freshly cleaved mica. These images were obtained via an Agilent 5500 AFM (Agilent Technologies) in alternating current mode using RTESP etched silicon probes (length: 125 µm, nominal force constant: 40 N/m, and resonance frequency: 275 kHz) (Veeco Instruments). While under ambient environmental conditions, the scan rate was held constant at 1 Hz. Each image (512x512 data points) was processed using Gwyddion version 2.7 software to remove artifacts and improve image quality by applying plane leveling, line correction and scar removal operations. This software was also used to extract height profiles, which allowed for the calculation of average feature heights. Multiple areas were imaged for each sample and while height, phase and amplitude images are collected simultaneously, only representative amplitude images are presented.

### Circular dichroism (CD)

CD spectra were obtained in the far UV region with a Jasco J-810 spectropolarimeter (Jasco Inc, Easton, MD). Aliquots of samples containing Aβ42 (25 µM) were placed in a 0.1 cm path-length quartz cuvette (Hellma) and were monitored in continuous scan mode (260–190 nm). The acquisition parameters were 50 nm/min with 8 s response time, 1 nm bandwidth and 0.1 nm data pitch, and data sets were averaged over three scans. All data were collected in duplicate. Spectra of appropriate blanks were subtracted from the data sets as indicated. The corrected, averaged spectra were smoothed using the ‘means-movement’ algorithm with a convolution width of 25 using the Jasco spectra analysis program.

### Seeding experiments

Monomeric Aβ42 (25 µM) was incubated alone or with different concentrations of fatty acids in a buffer containing 50 mM NaCl and 20 mM Tris pH 8.0. After 48 hr 10% (m/v) seeds of the incubated sample were withdrawn and mixed with fresh monomeric Aβ42 (25 µM) samples and incubated at 37°C under quiescent condition along with a control lacking seed. The rate of Aβ42 aggregation was monitored using ThT assay. Similar experiments were performed with isolated oligomers also.

### Gn-HCl denaturation experiments

The thermodynamic stability of the oligomers formed in the presence of fatty acids was determined by guanidine-HCl (GnHCl) denaturation. Aβ42 (25 µM) samples in the presence of specific concentrations of fatty acids (to generate 12–18mers or 4–5mers) were incubated at 37°C. Aliquots of the samples were taken after 48 h of incubation and tyrosine intrinsic fluorescence was measured using λ_ex_ 276 nm and scanning the emission spectrum (λ_em_) between 300–400 nm with a excitation/emission slit widths of 10/10 nm on a Cary Eclipse fluorescence spectrometer (Varian Inc). The sample was then subjected to denaturation by tritrating with 6M GnHCl stock solution into the fluorescence cuvette, at room temperature. Three scans were collected and averaged. Control spectra were measured by adding buffer without fatty acids to buffered 25 µM Aβ42 using the same volumes of GnHCl, which were subtracted from the sample spectra. The area under the curve of the blank-corrected spectra was plotted against the GnHCl concentration. The data were then normalized against the lowest and highest fluorescence intensity for the given fatty acid concentration. Three such blank-corrected spectra were averaged and are represented here. The resulting curve is fitted using the following Boltzmann equation;



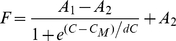
(Eq 2)where, *A1* and *A2* are constants, *C* is the concentration of the denaturant and *C_M_* is the mid-point of the curve that is considered as the concentration of ‘melting’. The data were processed using Origin 7.0.

## Supporting Information

Figure S1 Far-UV CD spectra of 25 µM Aβ42 incubations with NEFAs at concentrations well below the respective CMCs; 20 mM C9 (A), 10 mM C10 (B), 2 mM C11 (C) and 2 mM C12 (D). To data points are shown; initial 0 h point and the final 240 h point. Data were normalized as mentioned in Materials and Methods.
(TIF)

Figure S2 Guanidine denaturation of hGRN-A incubated with NEFAs as a negative control. Buffered (20 mM TrisHCl, 50 mM NaCl, pH 8.0) hGRN-A (15 µM) was incubated with 5 (○) or 20 mM C12 (▴) at 37 C.After 48 h, aliquots of the sample was subjected to GnHCl titration (using 6 M GnHCl as stock) that was monitored by tryptophan intrinsic fluorescence (λ_ex_ = 280 nm; λ_em_ = 340 nm). Data were normalized and fit using the same calculations as mentioned in Materials and Methods.
(TIF)

Figure S3 Incubation of 5 & 20 mM C12 with varying concentrations of Aβ42 ; lane (1–4) 5 mM C12 with 12.5, 25, 50, 75 µM Aβ42 respectively ; lane (5–8) 20 mM C12 with 12.5, 25, 50, 75 µM Aβ42 respectively.
(TIF)
