# Supplementary figures and images for: Non-Esterified Fatty Acids Generate Distinct Low-Molecular Weight Amyloid-β (Aβ42) Oligomers along Pathway Different from Fibril Formation
Source: PLoS One. 2011 Apr 19;6(4):e18759. doi: 10.1371/journal.pone.0018759 (PMC3079704; doi:10.1371/journal.pone.0018759)

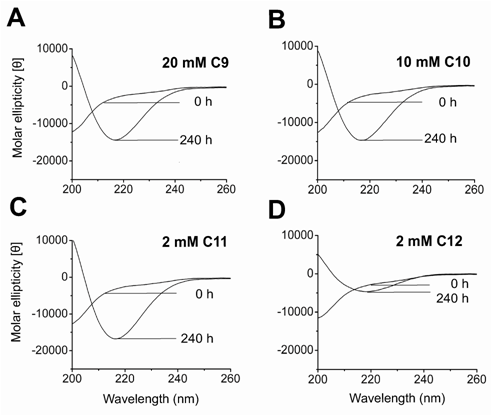

Supplement: Figure S1 — Far-UV CD spectra of 25 µM Aβ42 incubations with NEFAs at concentrations well below the respective CMCs; 20 mM C9 (A), 10 mM C10 (B), 2 mM C11 (C) and 2 mM C12 (D). To data points are shown; initial 0 h point and the final 240 h point. Data were normalized as mentioned in Materials and Methods. (TIF) [file pone.0018759.s001.tif]

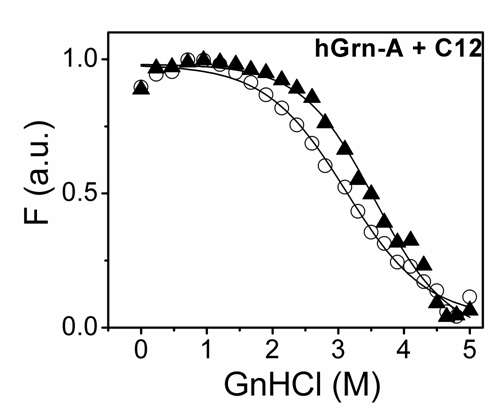

Supplement: Figure S2 — Guanidine denaturation of hGRN-A incubated with NEFAs as a negative control. Buffered (20 mM TrisHCl, 50 mM NaCl, pH 8.0) hGRN-A (15 µM) was incubated with 5 (○) or 20 mM C12 (▴) at 37 C.After 48 h, aliquots of the sample was subjected to GnHCl titration (using 6 M GnHCl as stock) that was monitored by tryptophan intrinsic fluorescence (λex = 280 nm; λem = 340 nm). Data were normalized and fit using the same calculations as mentioned in Materials and Methods. (TIF) [file pone.0018759.s002.tif]

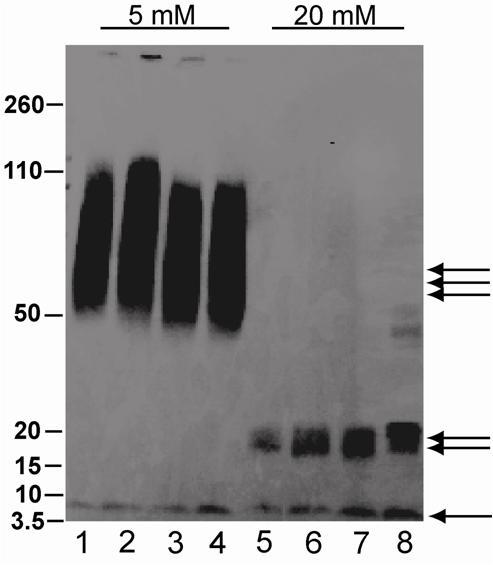

Supplement: Figure S3 — Incubation of 5 & 20 mM C12 with varying concentrations of Aβ42 ; lane (1–4) 5 mM C12 with 12.5, 25, 50, 75 µM Aβ42 respectively ; lane (5–8) 20 mM C12 with 12.5, 25, 50, 75 µM Aβ42 respectively. (TIF) [file pone.0018759.s003.tif]
